# Psychometric evaluation of an instrument measuring artificial intelligence utilization in decision-making domains of healthcare organizations

**DOI:** 10.1038/s41598-025-20753-9

**Published:** 2025-10-21

**Authors:** Zahra Zare, Mohsen Khosravi, Milad Ahmadi Marzaleh, Faride Sadat Jalali, Reyhane Izadi, Homeira Naseh

**Affiliations:** 1 Student Research Committee, Shiraz University of Medical Sciences, Shiraz, Iran; 2 Social Determinants of Health Research Center, Birjand University of Medical Sciences, Birjand, Iran; 3 Department of Health in Disasters and Emergencies, School of Health Management and Information Sciences, Shiraz University of Medical Sciences, Shiraz, Iran; 4 Health Human Resources Research Center, School of Health Management and Information Sciences, Shiraz University of Medical Sciences, Shiraz, Iran; 5 Student Research Committee, Birjand University of Medical Sciences, Birjand, Iran

**Keywords:** Organization and administration, Decision making, Delivery of health care, Learning health system, Artificial intelligence, Health care, Medical research

## Abstract

The decision-making process in healthcare services encounters numerous challenges. Artificial intelligence (AI) has significantly contributed to enhancing healthcare decision-making. There is a lack of validated instruments available in the literature to measure AI utilization across various healthcare domains. This study aimed to validate an instrument designed to assess the level of AI utilization across various healthcare domains within healthcare organizations. This study was conducted in Iran during the 2024–2025 period, utilizing a methodological design for the development of the study instrument. Initially, the authors formulated and constructed items for a preliminary questionnaire based on a previously published study within the relevant context. To ensure the instrument’s validity and reliability, a comprehensive evaluation was performed using multiple methods, including assessments of face validity, content validity, construct validity, and reliability analysis. The final version of the study instrument consisted of 12 items. The instrument demonstrated excellent validity and reliability. The average factor loading across the instrument’s items was 0.8, and the principal component accounted for 65.31% of the total variance. Additionally, both Cronbach’s alpha and the intraclass correlation coefficient (ICC) values were 0.95, indicating high internal consistency and reliability. Furthermore, the findings indicated that the level of AI utilization in Iran was predominantly low across most assessed items. The study presented a validated instrument for assessing AI implementation across healthcare decision-making domains. Further research is needed to develop specialized instruments for each decision-making domain to enhance data comprehensiveness.

**Supplementary Information:**

The online version contains supplementary material available at 10.1038/s41598-025-20753-9.

## Introduction

The health system policy and medical services revolve around the vital and highly important process of decision-making, which involves choices made by healthcare providers as well as shared decisions between patients and providers. These decisions are often made under conditions of uncertainty and highlight the importance of evidence-based guidelines that integrate considerations of quality of life and patient preferences^1^.

The decision-making process in healthcare services encounters numerous challenges. One of the primary difficulties is medical decision-making, which is inherently constrained by uncertainty and ambiguity. Another challenge is the need for effective evaluation in medical decision-making. Decision-makers must succinctly assess situations to make better choices^2^. Furthermore, patient preferences and participation in decision-making can influence the delivery of healthcare services. Some patients may prefer to delegate decisions to the physician, while others may wish to take a more active role in the decision-making process. Physicians should be aware of these preferences and adopt a patient-centered decision-making approach^3^.

Artificial intelligence (AI) refers to computer programs that perform tasks requiring intelligence and analysis, such as problem-solving and learning processes. AI includes machine learning, which is the science of enabling computers to learn independently from data and continuously adapt to new data without explicit programming^4^. Machine learning and deep learning are two main branches of AI utilized in medical technologies. Machine learning is a subset of AI that can automatically learn and improve from experience without explicit programming. Machine learning algorithms rely on characteristic features, with some predominant methods including Artificial Neural Networks (ANN), Random Forest (RF), Support Vector Machines (SVM), and Decision Trees (DT). Deep learning, a subset of machine learning, can solve complex patterns through representation learning. Some common deep learning methods include Convolutional Neural Networks (CNN), Recurrent Neural Networks (RNN), and Long Short-Term Memory (LSTM)^5–8^.

The literature indicates the applicability of AI in three decision-making domains within the healthcare service delivery process: shared decision-making, clinical decision-making, and organizational decision-making^9^. AI has made a significant contribution to healthcare decision-making. These contributions include enhancing clinical decision-making processes, predicting outcomes, and facilitating personalized and customized information for patients^10,11^. It has also been demonstrated that AI algorithms perform as well as, or even better than, humans in analyzing medical images or correlating symptoms and biomarkers from electronic medical records with diseases. Furthermore, AI has been utilized to develop adaptive and personalized educational software that can tailor educational content, pace, feedback, and assessment to individual needs. AI is also employed to increase patient autonomy by enabling patients to receive personalized treatment information and supporting ethical treatment decisions^9^.

In the field of clinical decision-making, AI has demonstrated remarkable capabilities in predicting and classifying diagnoses, as well as providing recommendations and insights^12,13^. Knowledge-based clinical decision support systems have the potential to enhance physician performance^14,15^. Research literature indicates that AI systems are applicable in various medical imaging use cases, such as mitosis detection in breast cancer histopathology images, skin cancer classification, diabetic retinopathy diagnosis, and prediction of cardiovascular risk factors^16–19^. These studies highlight the potential of AI systems to assist healthcare providers in improving diagnosis and delivering personalized information. Moreover, AI has also been presented to possess the ability to predict suicide risk^20^.

This study aimed to develop a valid instrument and assess the level of AI utilization across healthcare domains within healthcare organizations in the Iranian healthcare system. At the time this study was conducted, no previous research with a similar objective and methodology was found in the literature, highlighting the originality of the present study. The collection and design of items related to the use of AI in the decision-making areas, structured as a checklist with appropriate validity and reliability, followed by field evaluation of AI utilization levels in these domains within healthcare organizations, can yield tangible and significant benefits for policymakers, organizational managers, researchers, and other stakeholders. Policymakers and healthcare organizational managers, by identifying and assessing the status of their respective organizations regarding the level of AI utilization in decision-making through a psychometrically validated checklist, can facilitate targeted and planned adoption of this technology in the healthcare decision-making system. Moreover, through continuous evaluation and feedback on the extent of AI deployment in organizational decision-making, a foundation can be established for the ongoing, strategic, and systematic expansion of AI utilization. This approach will ultimately lead to substantial and progressive development of healthcare organizations within the decision-making domain.

## Methods

This study was conducted in Iran during the 2024–2025 period, utilizing a methodological design for the development of the study instrument. The researchers aimed to design, evaluate, and validate a tool intended to measure the extent of AI usage in decision-making processes within healthcare organizations. Initially, the authors developed and constructed items for a preliminary questionnaire, informed by a previously published study relevant to the field. To ensure the instrument’s validity and reliability, a thorough evaluation was performed employing multiple methods, including assessments of face validity, content validity, construct validity, and reliability analysis. The study’s data reporting adhered to the STROBE (Strengthening the Reporting of Observational Studies in Epidemiology) guidelines^21^.

### Research question

The research question was developed as: “What is the level of AI utilization in healthcare domains of healthcare organizations?”.

### Study sample and sampling method

In this study, purposive and convenience sampling techniques were employed. To evaluate the quantitative face validity, the developed items were presented to a panel of 10 experts and 10 stakeholders, comprising professors and professionals in healthcare management, as well as employees from various organizations within the Iranian healthcare sector. Furthermore, input from four experts in the Persian language was incorporated to ensure the linguistic precision of the instrument’s items. During the content validity phase, 20 experts affiliated with the Iranian health system, including professors and health management professionals, participated in the assessment. For construct validity, a sample size equivalent to ten times the number of instrument items (120 individuals), composed of employees from organizations associated with the health system, was utilized. Finally, in the assessment of reliability, a group of 30 employees from health system-affiliated organizations was examined.

### Phase one: item construction

In this phase of the study, the design of the questionnaire items for psychometric evaluation was informed by findings from a literature review conducted by the current authors, published in 2024 (details provided in Table 1). This literature review systematically examined review articles published in English between 2000 and 2024, sourced from databases including PubMed, Scopus, ProQuest, and Cochrane. Following a rigorous quality assessment of the selected studies using the Critical Appraisal Skills Programme (CASP) checklist, final studies were chosen for inclusion. Subsequently, thematic analysis was applied to synthesize and present the findings. Further details are available in the full article published on the journal’s website. To clarify, the CASP checklist is a structured tool used to assess the quality and validity of studies systematically, ensuring a critical appraisal of research evidence. On the other hand, the thematic analysis is a qualitative method that involves identifying and interpreting patterns or themes within the data through a systematic process, which facilitates rich and organized presentation of insights^[9]^.

**Table 1:** Application domains of AI in healthcare decision-makings^9^.

Dimension	Sub-dimension
Clinical decision-making	Remote monitoring
	Computerized interpretation of graphs
	Facilitating diagnosis and prognosis
Organizational decision-making	Forecasting administrative and quality indicators
	Offering cost-effective solutions for time and resource management
Shared decision-making	Provision of personalized and customized information
	Enabling patient self-management
	Enhancing patient medication adherence

### Phase two: validity

Instrument validity generally encompasses three primary types: face validity, content validity, and construct validity. Collectively, these forms of validity provide robust evidence that the instrument accurately measures the intended construct. Establishing validity is a crucial aspect of the instrument development process, ensuring that the measure is appropriate, relevant, and meaningful for its intended purpose and target population^22^.

#### Face validity

Face validity pertains to a subjective evaluation of a research instrument’s relevance, format, readability, clarity, and appropriateness for its intended audience. It represents the most fundamental form of validity, primarily grounded in the instrument’s appearance and overall presentation^23–25^.

In this phase of the study, four experts proficient in the local language (Persian) conducted a thorough review of the instrument items to identify and rectify any inappropriate wording and grammatical errors. During the process, the instrument items were revised in accordance with expert recommendations to improve clarity, readability, and to correct grammatical errors. Following this, a panel consisting of 10 professors and professionals in healthcare management, as well as 10 employees from various organizations within the Iranian healthcare sector, was engaged.

Subsequently, the ‘impact score’ method was applied to eliminate unsuitable items and quantitatively assess the validity of each item within the preliminary instrument. The impact score in face validity evaluations measures the relative importance of individual items within a measurement tool. The item impact score was computed according to the following formula^[25]^:

Item Impact Score = Frequency (percentage) × Importance.

In this context, “frequency” refers to the percentage of respondents who rated an item as important on a Likert scale, while “importance” corresponds to the mean rating assigned to that item. Typically, an impact score of 1.5 or greater is regarded as the minimum acceptable threshold for retaining an item within the instrument. This criterion signifies that at least 50% of respondents evaluated the item as important, providing a rating of 4 or 5 on a 5-point scale^25^.

The inclusion criteria for experts participating in this stage were as follows:

- Possession of familiarity with the concept of AI and its application within healthcare.

For experts proficient in the local language (Persian):

- Holding an academic degree specializing in the local language.

For professionals in healthcare management:

- Holding a doctoral degree (Ph.D.) in healthcare management or health policy.

For employees within healthcare organizations:

- Having practical experience in the utilization of AI for healthcare decision-making processes.

The exclusion criterion was defined as:

- Inability to complete the questionnaire in either physical or digital formats.

#### Content validity

Content validity denotes the extent to which the selected items comprehensively and accurately represent the construct under measurement. The evaluation of content validity generally involves assembling a panel of experts who assess each item’s relevance and representativeness concerning the specific content domain being examined^26^.

In this study, content validity was evaluated utilizing Lawshe’s methodology. This method involves distributing questionnaires to a panel of experts, who assess the necessity and importance of each item within the instrument. Specifically, the Content Validity Ratio (CVR) and the Content Validity Index (CVI) were computed for each item to quantify the extent of expert agreement regarding the essentiality and relevance of the items. The CVR is computed using the following formula^27^:

$$\text{CVR=(Ne-N/2)/(N/2)}$$

Where:

- Ne​ = number of experts who rated an item as “essential”.
- N = total number of experts.

An item with a CVR of 0.42 or above is considered relevant for inclusion^28^. Moreover, an item with a CVI of 0.7 or higher is considered relevant for inclusion, whereas items with CVI values below this threshold are typically recommended for removal^29^. During this phase of the study, items with borderline CVI scores were either revised or consolidated with other items, adhering to evidence-based guidelines. The CVI is calculated using the following formula^25^:

$$\text{CVI = Number of experts rating an item as relevant/Total number of experts}$$

A total of 20 professors and professionals specializing in health management participated in this stage. The inclusion criteria for expert participation were as follows:

- Possession of familiarity with the concept of AI and its application within healthcare.
- Holding a doctoral degree (Ph.D.) in healthcare management or health policy.

The exclusion criterion was defined as:

- Inability to complete the questionnaire in either physical or digital formats.

#### Construct validity

Construct validity refers to the degree to which an instrument or test accurately measures the theoretical construct it is intended to assess. This type of validity is established by examining the relationships between the instrument and other measures of the same construct (convergent validity), as well as its associations with different, theoretically distinct constructs (discriminant validity), in alignment with established theoretical frameworks. Construct validity ensures that the measure behaves as expected according to theory, providing confidence that the instrument truly reflects the intended abstract concept rather than unrelated constructs^30^.

In the present study, exploratory factor analysis (EFA) was utilized to establish the construct validity of the instrument through the principal component analysis technique. EFA is a statistical method that enables researchers to condense a large set of variables or items into a smaller number of latent factors, thereby clarifying the underlying structure of the relationships among these variables. This technique offers evidence of construct validity by illuminating the internal composition of the instrument and the theoretical constructs it is intended to measure^31^.

The exploratory factor analysis (EFA) procedure typically commences with the selection of items grounded in theoretical justification and expert consultation. This is followed by statistical analysis to ascertain the extent to which these items load onto distinct factors. Factor loadings represent the magnitude of the association between each item and a specific factor; a loading exceeding 0.30 is commonly regarded as indicative of a moderate correlation^31^. In this study, a sample of 120 employees from healthcare organizations was employed, which was considered appropriate given the 12 items included in the instrument at this stage. This sample size conforms to the widely accepted guideline recommending a minimum of 5 to 10 participants per variable^32,33^.

Prior to conducting exploratory factor analysis (EFA), the suitability of the data for factor analysis was assessed using the Kaiser–Meyer–Olkin (KMO) measure of sampling adequacy and Bartlett’s test of sphericity. The KMO statistic quantifies the proportion of variance among variables that may be common variance, with values ranging from 0 to 1. A KMO value above 0.5 is deemed acceptable for factor analysis, while values exceeding 0.8 indicate a high degree of suitability^34^. Bartlett’s test of sphericity evaluates whether the correlation matrix significantly differs from an identity matrix, thus determining if the variables exhibit sufficient correlation for factor analysis. A statistically significant result (typically *p* < 0.05) suggests that the data are appropriate for factor analysis^35,36^. All statistical analyses in this stage were performed using IBM SPSS version 27.0.1.

Upon confirmation of data suitability through the results of the Kaiser–Meyer–Olkin (KMO) measure and Bartlett’s test of sphericity, principal components were extracted and summarized using a Varimax rotated component matrix. During the process, the number of final components was determined based on the eigenvalues observed in the scree plot. Two authors independently reviewed the distribution of each item across the extracted components based on factor loadings and proposed labels for each component according to the thematic content of the items. Any discrepancies between the two authors were resolved through consultation with a third author. Moreover, during the data analysis process, missing data were addressed by calculating the mean of the available values for each item and imputing the missing values with the corresponding item mean.

The inclusion criterion for participants in this phase was:

- Practical experience in utilizing AI for healthcare decision-making.

The exclusion criterion was:

- Inability to complete the questionnaire in either physical or digital format.

### Phase three: reliability

Reliability pertains to the precision and consistency of the data obtained^22^. To assess the reliability of the study instrument, two statistical measures were employed: Cronbach’s alpha and the intraclass correlation coefficient (ICC).

Cronbach’s alpha (α) is a statistical measure of internal consistency, indicating the extent to which a set of items are interrelated and collectively form a cohesive scale. A Cronbach’s alpha value above 0.70 is generally considered acceptable, reflecting adequate homogeneity among the items. Values exceeding 0.80 suggest good reliability, while those above 0.90 indicate excellent internal consistency^37^. Conversely, the ICC serves as a reliability metric that assesses both the degree of correlation and agreement among measurements. ICC values below 0.5 are indicative of poor reliability; values between 0.5 and 0.75 denote moderate reliability; values from 0.75 to 0.9 represent good reliability; and values above 0.9 signify excellent reliability^37^. For this phase of the study, a sample comprising 30 employees from healthcare organizations was employed to assess the reliability of the instrument, consistent with the research published by Bujang et al. (2024) that identifies this number as the minimum sample size required for such analyses^38^.

The inclusion criterion for participants in this phase was:

- Practical experience in utilizing AI for healthcare decision-making.

The exclusion criterion was:

- Inability to complete the questionnaire in either physical or digital format.

### Ethical considerations

All study data will be retained and securely stored for a minimum period of one year following the publication of the article. The data analysis at all stages was performed by researchers who declared no conflicts of interest concerning the study topic or the organizations involved. Furthermore, in accordance with established ethical standards for research employing such methodologies, the analysis of the collected data was carried out in a manner that preserved participant anonymity and ensured strict confidentiality of all information. Informed consent was obtained from all participants prior to their inclusion in the study. Additionally, consistent with ethical principles, participants were afforded the right to withdraw from the study at any time without incurring any penalties or loss of benefits.

## Results

The findings corresponding to each phase of the study are detailed in the subsequent sections.

### Item construction

Upon completion of the item construction process, a questionnaire consisting of 15 items was developed to undergo psychometric evaluation. The initial items of the study instrument are presented below:

- Q1: Is artificial intelligence technology utilized for managing and analyzing clinical data?
- Q2: Is artificial intelligence technology employed to predict patients’ need for hospitalization?
- Q3: Is artificial intelligence technology used to identify disease patterns within clinical data?
- Q4: Is artificial intelligence technology applied to enhance the quality and accuracy of diagnostic tests?
- Q5: Is artificial intelligence technology utilized in the development of new drugs and treatments?
- Q6: Is artificial intelligence technology used to analyze clinical data for improving managerial decision-making processes?
- Q7: Is artificial intelligence technology employed to forecast trends related to healthcare service demand?
- Q8: Is artificial intelligence technology applied to optimize scheduling and resource allocation in hospitals?
- Q9: Is artificial intelligence technology used for assessing and managing financial risks in hospitals?
- Q10: Is artificial intelligence technology utilized to facilitate staff education and professional development?
- Q11: Is artificial intelligence technology employed to provide medical consultation services based on patients’ medical histories?
- Q12: Is artificial intelligence technology used to create personalized care plans tailored to the specific needs of patients?
- Q13: Is artificial intelligence technology applied to analyze patients’ health behaviors and offer appropriate recommendations?
- Q14: Is artificial intelligence technology used to facilitate patients’ access to their medical information?
- Q15: Is artificial intelligence technology utilized to assess and predict patients’ needs?

### Validity

#### Face validity

**Fig. 1:**
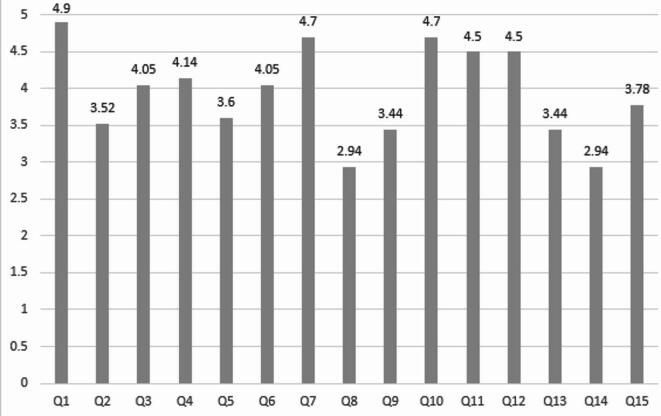
Impact scores for each of the initial study instrument items.

Following the completion of the qualitative face validity assessment and the revision of the study instrument by experts in the Persian language, the quantitative face validity was evaluated using the impact score method, as previously described in the methodology section. This analysis demonstrated that all initial instrument items achieved impact scores within an acceptable range, spanning from 2.94 to 4.9 (Fig. 1). During this stage of the study, no items were deleted or combined with other items.

#### Content validity

**Fig. 2:**
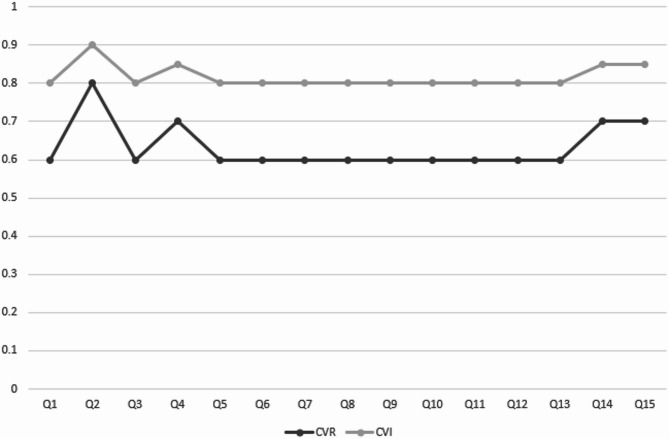
CVR and CVI scores for each of the initial study instrument items.

Upon completion of the content validity assessment, all items of the instrument demonstrated acceptable CVR and CVI scores. The CVR scores ranged from a minimum of 0.6 to a maximum of 0.8, while the CVI scores ranged from 0.8 to 0.9. (Fig. 2). However, the experts recommended integrating three items (Q2, Q3, and Q13) with other items in the instrument, such as Q7, Q15, Q1, and Q10.

#### Construct validity

As previously indicated in the manuscript, at this stage of the study, a sample of 120 employees from multiple types of healthcare organizations was utilized. The mean age of the participants was approximately 35 years. Among them, 52.2% were employed in hospitals, 22.5% worked at universities, and the remaining participants were employed in other types of healthcare centers. Regarding educational qualifications, 33.94% held a Master of Science (MSc) degree, 45.76% a Bachelor of Science (BSc) degree, 17.79% a Doctor of Philosophy (Ph.D.) degree, 4.2% were medical specialists, and the remainder were Doctor of Medicine (M.D.) holders. The participants’ fields of education encompassed multiple disciplines, predominantly including health economics, health informatics, healthcare services management, public health, medicine, and nursing.

As presented in Table 2, the results of the KMO measure and Bartlett’s test of sphericity for the study instrument data indicated that the KMO value reflected moderate sampling adequacy, while the significant Chi-Square statistic demonstrated the presence of substantial correlations among the variables. Consequently, conducting factor analysis on this dataset was deemed appropriate.

**Table 2:** KMO and Bartlett`s test results.

KMO measure of sampling accuracy		0.914
Bartlett`s test of sphericity	Approx. Chi-Square	1282.695
	df	66
	Sig.	0.000

**Fig. 3:**
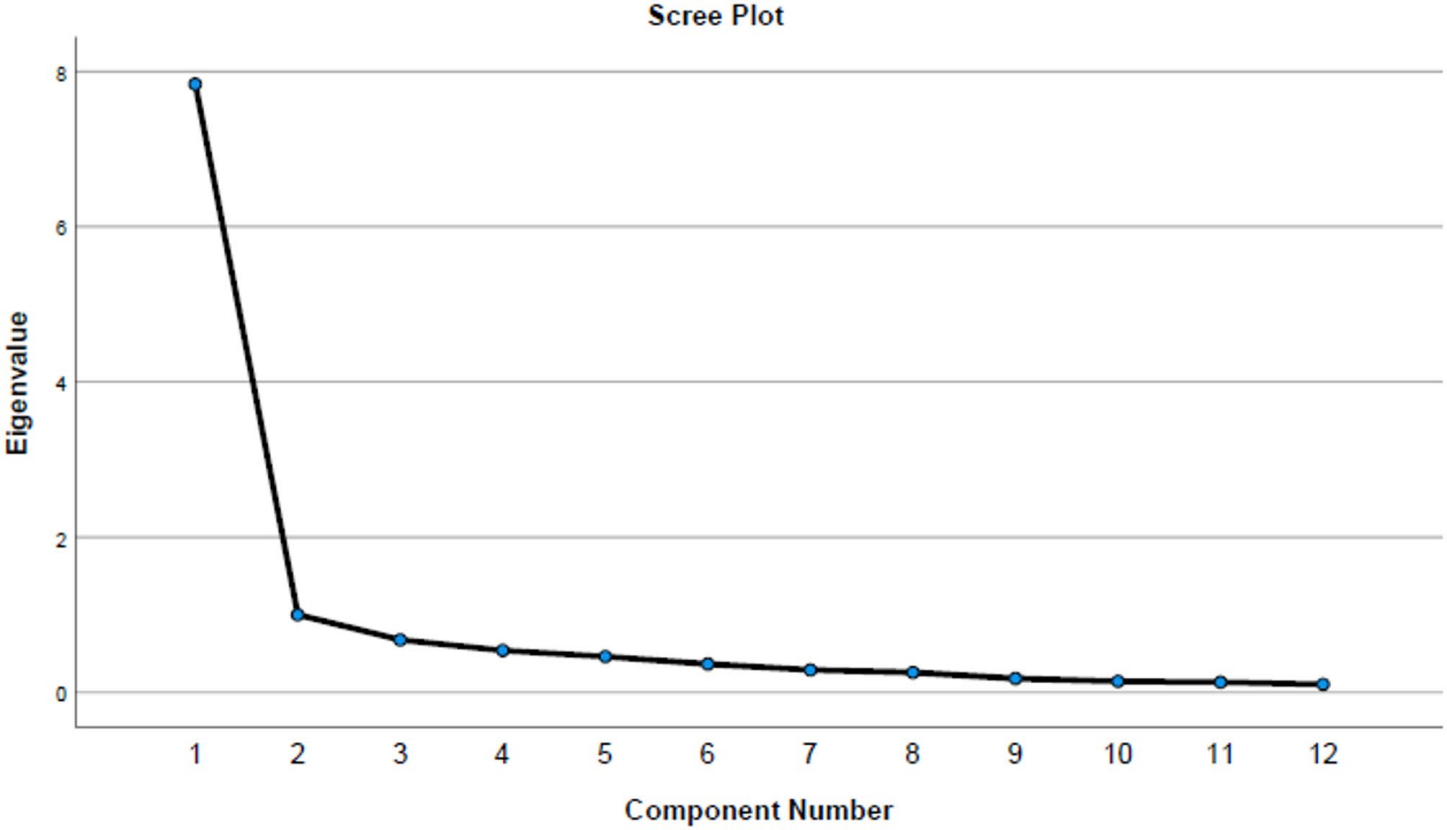
Scree plot.

As illustrated in Fig. 3, the eigenvalues, arranged in descending order from left to right, identify the number of significant factors by pinpointing the location where the curve levels off. In this context, the rotated Varimax component matrix revealed that the items of the study instrument load predominantly onto a single major component. The average factor loading across the instrument’s items was 0.8, signifying strong associations between the items and the extracted factor. Furthermore, this principal component accounted for 65.31% of the total variance. Detailed factor loadings for each item are provided in Table 3.

**Table 3:** Factor loadings of the study instrument items.

Item	Q1	Q4	Q5	Q6	Q7	Q8	Q9	Q10	Q11	Q12	Q14	Q15
Factor loading	0.757	0.786	0.747	0.812	0.818	0.870	0.795	0.878	0.803	0.802	0.819	0.801

### Reliability

Following the reliability analysis of the study instrument, which had been previously validated through earlier stages of the research, the instrument demonstrated high reliability with both Cronbach’s alpha and ICC values of 0.95. The Cronbach’s alpha value confirms the instrument’s consistency in measuring the extent of AI utilization within decision-making processes in healthcare organizations. Concurrently, the ICC value indicates strong inter-item correlations, reflecting that the items effectively measure the underlying constructs. The finalized version of the study instrument consisted of a 12-item, five-point Likert scale questionnaire, as detailed in Appendix 1 (Study Instrument).

## Discussion

**Fig. 4:**
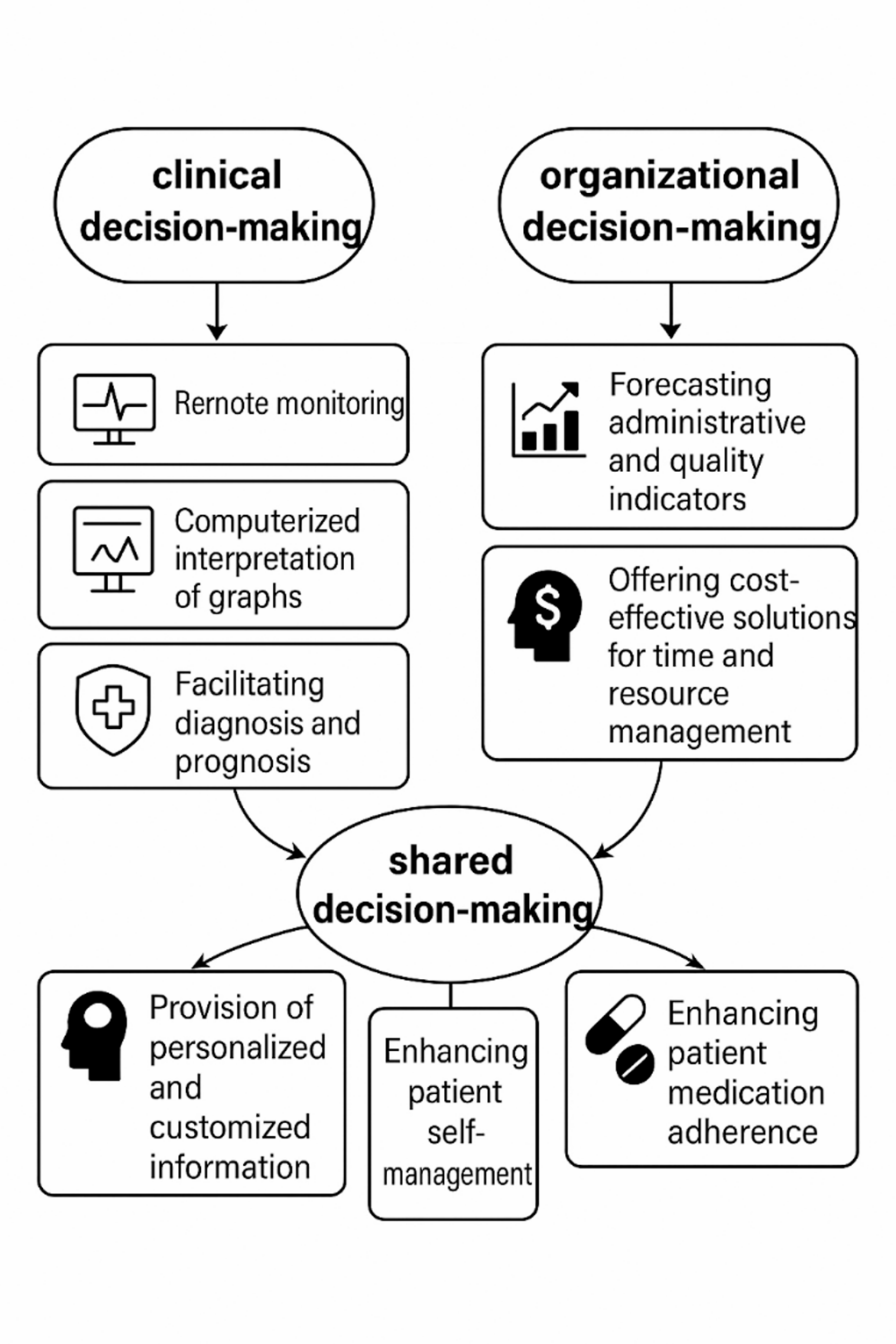
Description of the final items of the study instrument.

As indicated in the study results, the final version of the study instrument comprised 12 items and demonstrated a high degree of validity and reliability. In this regard, each of the three themes—clinical decision-making, organizational decision-making, and shared decision-making—that formed the foundation of this study was equally represented in the final version of the study instrument, with each theme comprising four items. To enhance clarity and readability for the audience, the description of the final items of the study instrument is delineated in Fig. 4.

As there have been no prior studies at the international level within this context that evaluate an instrument specifically designed to assess the level of AI utilization in healthcare organizations, and given that most existing instruments at the time of this study had primarily focused on individuals’ attitudes towards AI, this section of the study concentrated on the extent of AI utilization in Iran as determined by the study instrument^39–44^. Additionally, it aimed to address the primary challenges and potential solutions for AI implementation in Iran, as well as in other countries with similar contexts.

Although there are lack of studies evaluating instruments to assess AI utilization levels in healthcare organizations globally, one research in similar context was found. A study in Saudi Arabia examined AI use in healthcare decision-making among caregivers, finding that 75%—mainly nurses—reported using AI. The study identified significant correlations between AI utilization and factors such as age, nationality, specialty, experience, and training location, with more experienced and internationally trained caregivers reporting higher usage^45^. In contrast, our study found uniformly low levels of AI utilization across all domains among participants, precluding similar correlation analysis.

The comparatively advanced AI utilization in Saudi Arabia may stem from significant government initiatives aimed at positioning the country as a global AI hub, particularly in healthcare. As part of Vision 2030, the Saudi government prioritizes AI to enhance healthcare delivery through early disease detection, personalized treatment, predictive epidemiology, remote monitoring, chronic disease management, and resource optimization^46,47^. This strategy is supported by regulatory frameworks, public-private partnerships, education and training programs, innovation centers, research institutions, and funding incentives^46^. Consequently, AI applications are actively being developed and deployed in emergency medicine triage, telemedicine, remote patient monitoring, and healthcare management, underpinned by substantial investments in healthcare technology and digital infrastructure^46–48^.

The study results demonstrated that the level of AI utilization within healthcare organizations in the Iranian healthcare system was found to be markedly low, despite the existence of a considerable number of studies conducted in this context, particularly those assessing individuals’ attitudes towards AI in healthcare settings^39–41^. This finding holds significant implications for healthcare policymakers and managers in Iran, highlighting the inadequate performance of healthcare organizations in integrating AI technologies. In this regard, the content of the study instrument may serve as a valuable resource by identifying the specific domains within healthcare where AI implementation can be effectively incorporated.

The literature has identified several factors contributing to the limited utilization of AI in Iran’s healthcare system. Key challenges include inadequate information systems, lack of sustainable financing, and deficiencies in executive infrastructure^49^. Public resistance, stemming from low awareness of AI benefits and concerns regarding justice, accountability, and service quality, further hinders adoption^49^. Additionally, international sanctions have restricted the import of essential equipment, increasing costs and delaying AI development^50^. Weak governance, absence of strategic policies, ineffective oversight, and limited stakeholder collaboration also obstruct the implementation of AI-based services, including telemedicine^51^.

The literature has also identified several facilitators of AI adoption in the Iranian healthcare system. In this regard, transparent governance and robust regulatory frameworks build trust by addressing data security, privacy, and accountability concerns^52^. Moreover, moderate to positive knowledge and acceptance of AI among physicians and nurses, particularly when supported by education and training, further promote adoption^50^.

## Limitations and implications

The study had a limitation to address. Given the sample size available to the authors and the constraints of time and resources, it was not possible to measure the extent of the impact of AI utilization on improving the performance of decision-making domains in healthcare services; this issue can be an implication for future researchers within the context, suggesting them to conduct studies with such aim. Another limitation of the study was the authors’ limited time and resources, which constrained the breadth and comprehensiveness of the instrument’s items. In this regard, the conduction of the study and its sample was limited to a single country (Iran). Consequently, the perspectives of international experts were not incorporated, as accessing them was considered significantly challenging. Therefore, further research is necessary to develop specialized instruments that incorporate international expert and sample input across diverse regions and cultures and address each healthcare decision-making domain to provide more comprehensive data within this context. Utilizing different analytical approaches, such as confirmatory factor analysis, in future research to provide further insights regarding the data could be another implication for future researchers.

The study had some implications for the beneficiaries to address. In this regard, to the best of the authors’ knowledge, the results of this study represent one of the first, if not the very first, publications to validly and reliably identify the decision-making domains within healthcare where AI has the potential for application. In this regard, this study offers valuable data for healthcare policymakers and administrators globally who seek to implement AI within their respective healthcare organizations. The findings can be used to develop strategic and operational plans for AI integration, as well as to periodically assess the level of AI adoption within their institutions using the study’s evaluation instrument. The study also offers valuable insights for the Iranian healthcare system and countries with similar sociopolitical contexts by identifying barriers and facilitators of AI adoption.

## Conclusion

The study introduced a novel, validated instrument identifying multiple areas for AI implementation within the decision-making domains of healthcare organizations. This tool has the potential to serve as a foundational step for the validation of an international instrument that is cross-culturally validated across diverse regions, which healthcare institutions worldwide can employ to plan the adoption of AI across various organizational decision-making processes. Additionally, the study highlighted the notably low level of AI adoption in the Iranian healthcare system and identified key barriers and facilitators relevant to stakeholders. Further research is warranted to develop specialized instruments for each healthcare decision-making domain to provide more comprehensive data within this context.

## Supplementary Information

Below is the link to the electronic supplementary material.

Supplementary Material 1

Supplementary Material 2

## Data Availability

The research data is available upon contacting the corresponding authors of the paper.
