# Supplementary material for: Psychometric evaluation of an instrument measuring artificial intelligence utilization in decision-making domains of healthcare organizations
Source: Sci Rep. 2025 Oct 21;15:36698. doi: 10.1038/s41598-025-20753-9 (PMC12540851; doi:10.1038/s41598-025-20753-9)
Supplement: Supplementary file 1 — Supplementary Material 1 [file 41598_2025_20753_MOESM1_ESM.docx]

**Appendix 1. Study instrument.**

| **No.** | **Question** | **Always** | **Most of the Time** | **Usually** | **Rarely** | **Never** |
| --- | --- | --- | --- | --- | --- | --- |
| In my workplace… | | | | | | |
| 1 | Artificial intelligence technology is used for managing, pattern recognition, and analysis of clinical data. |  |  |  |  |  |
| 2 | Artificial intelligence technology is used to improve the quality and accuracy of diagnostic tests. |  |  |  |  |  |
| 3 | Artificial intelligence technology is used for the development of new drugs and treatments. |  |  |  |  |  |
| 4 | Artificial intelligence technology is used to analyze clinical data to enhance managerial decision-making processes. |  |  |  |  |  |
| 5 | Artificial intelligence technology is used to predict trends related to service demand. |  |  |  |  |  |
| 6 | Artificial intelligence technology is used to optimize scheduling and resource allocation. |  |  |  |  |  |
| 7 | Artificial intelligence technology is used to assess and manage financial risks. |  |  |  |  |  |
| 8 | Artificial intelligence technology is used to facilitate staff training and professional development. |  |  |  |  |  |
| 9 | Artificial intelligence technology is used to provide medical consultation services based on patient medical records. |  |  |  |  |  |
| 10 | Artificial intelligence technology is used to create personalized care plans based on specific patient needs. |  |  |  |  |  |
| 11 | Artificial intelligence technology is used to facilitate patients’ access to their medical information. |  |  |  |  |  |
| 12 | Artificial intelligence technology is used to assess and predict the needs of visitors. |  |  |  |  |  |
